# Chemical composition and larvicidal activities of essential oil of *Cinnamomum camphora* (L.) leaf against *Anopheles stephensi*

**DOI:** 10.1590/0037-8682-0211-2019

**Published:** 2020-01-27

**Authors:** Yite Xu, Jie Qin, Pan Wang, Qiuxia Li, Shasha Yu, Yanling Zhang, Ying Wang

**Affiliations:** 1 Department of Tropical Medicine, College of Preventive Medicine, Army Medical University, Chongqing 400038, China.; 2 The 10th Squadron, The 3rd Brigade, College of Basic Medicine, Army Medical University, Chongqing 400038, China.; 3 Department of Neurology, Southwest Hospital, Army Medical University, Chongqing 400038, China.

**Keywords:** Essential oil, *Cinnamomum camphora* (L.), Larvicide, Anopheles stephensi, Chemical composition.

## Abstract

**INTRODUCTION:**

*Anopheles stephensi* is the main malaria vector in Southeast Asia. Recently, plant-sourced larvicides are attracting great interests.

**METHODS:**

The essential oil was extracted from the leaf of *Cinnamomum camphora* (L.), and a bioassay was conducted to determine the larvicidal efficacy. The chemical composition of the essential oil was determined by GC-MS analysis.

**RESULTS:**

The oil showed strong, dose-dependent larvicidal activities. The onset of larvicidal efficiency was rapid. The LC50 and LC95 were determined as 0.146% and 1.057% at 1 h, 0.031% and 0.237% at 12 h, 0.026% and 0.128% at 24 h, respectively. The oil contains 32 compounds.

**CONCLUSIONS:**

The essential oil of *C. camphora* leaf has an excellent larvicidal potential for the control of *A. stephensi*.

Mosquitos can transmit various infectious diseases, such as malaria, dengue, Zika, and chikungunya, etc^1^. *Anopheles stephensi* is the most important malaria vector in Southeast Asia. Mosquito vector control is considered as an efficient strategy to block the transmission of mosquito-borne diseases. The conflict between humans and mosquitoes has been intensified during the past years. Chemical insecticides have been used widely for decades for the control of mosquitoes, offering various advantages, and have yielded a great contribution to improvements in human survival and living standards. However, unreasonable long-term abuse has begun to lead to the destruction of the ecological balance, pollution of environment and development of resistance^2^ ^,^ ^3^. Besides, the harmful effects of chemical insecticides on humans is also of concern. It was shown that prolonged exposure to insecticides can affect the nervous system, blood system, reproductive system, digestive system, and endocrine system^3^. Therefore, there is an urgent need for new environmentally friendly measures and strategies for mosquito control^1^. 

Biological pesticides may be suitable substitutes for chemical pesticides; among them, volatile plant oils appear one of the best group of candidates. An increasing number of studies have focused on botanicals containing active phytochemicals with pesticidal potential. Camphor trees have a camphor-like aroma and are commonly distributed in the South and Southwest of the Yangtze River in China. The camphor tree is a medicinal plants and is of great significance in Chinese traditional medicine. For pest control, most studies have focused on the insecticidal and repellent activity of essential oil from *Cinnamomum camphora* (L.)^4^. Plant-based repellents have been used for generations in traditional practice as a personal protective measure against host-seeking mosquitoes. The essential oil of camphor tree leaves has been found to have a certain degree of mosquito repellence. Natural products, such as camphor and lavender oils, can be also developed as larvicides against pests such as *Lucilia sericata* ^5^. There are few studies on the larvicidal activity of the essential oil of camphor leaf against *A. stephensi* with the exception of one paper reporting limited information^6^. 

In the present study, we investigated the larvicidal activity of the essential oil from leaf of *C. camphora* (L.) against *A. stephensi.* In addition, the chemical components of the oil were analyzed by using gas chromatography-mass spectrometry, to explore the likely killing mechanisms.

First, the essential oil was extracted from fresh leaves of *C. camphora* (L.) by hydrodistillation. The fresh leaves were washed, cut into pieces, and placed into a 500 mL flask. Distilled water was then added, to make a leaf:water ratio of 1:14 (w/w). The flask was connected with the condenser tube and placed on the essential oil extraction device. The flask was heated and the contents were kept at a low boil for 70 min. The upper layer (essential oil) was collected into a tube; the volume obtained was measured and stored at 4^o^C until use. Using such a method, a volatile oil with a strong odor was extracted from *C. camphora* (L.) with a yield of 0.08 mL oil per gram of leaves.

Then, a bioassay was conducted to determine the killing efficiency of the essential oil on *A. stephensi* larvae. The early stage of the fourth instar larvae of *A. stephensi* were divided into six groups, with each group containing 50 larvae and 100 mL of larval rearing water. Triplicate tests were set up in each group. A gradient of essential oil concentrations (0%, 0.01%, 0.03%, 0.06%, 0.12%, and 0.24%) was tested among the groups. Larval deaths were counted and dead larva were removed at 0.5 h, 1 h, 2 h, 3 h, 4 h, 5 h, 6 h, 12 h, 24 h, 48 h, 72 h, and 96 h after essential oil treatment. Then, the accumulated mortality was calculated and corrected according to the Abbott’s formula:

$$Corrected\;mortality\;\left(\%\right)=\frac{\left(Mobs-Mcontrol\right)}{\left(100-Mcontrol\right)}x\;100.$$

The Probit value of the mortality was obtained from the Probit table. Moreover, linear regression analysis was conducted by using GraphPad Prism 5.01 to investigate the relationship between the Probit value and logarithm of the oil concentration. Finally, LC50 and LC95 were calculated from the formulae obtained from the linear regression analysis. Based on the bioassay results, the larvicidal efficacy of the volatile oil is dose dependent and the accumulated morality of the fourth instar larvae increased with an increase in incubation time and oil concentration. The onset of larvicidal efficiency was early, and peaked at 30 min and sub-peaked at 3 h (Figure 1). In addition, there was an ideal linear relationship between the Probit value of the accumulated mortalities and log_10_(oil concentration) (Figure 2). The LC50 and LC95, calculated according to the obtained formula as y=1.918x+10.44 at 1 h, y=1.859x+11.53 at 12 h, and y=2.415x+13.64 at 24 h, were 0.146% (ν/ν) and 1.057% at 1 h, 0.031% and 0.237% at 12 h, and 0.026% and 0.128% at 24 h, respectively.

**FIGURE 1:**
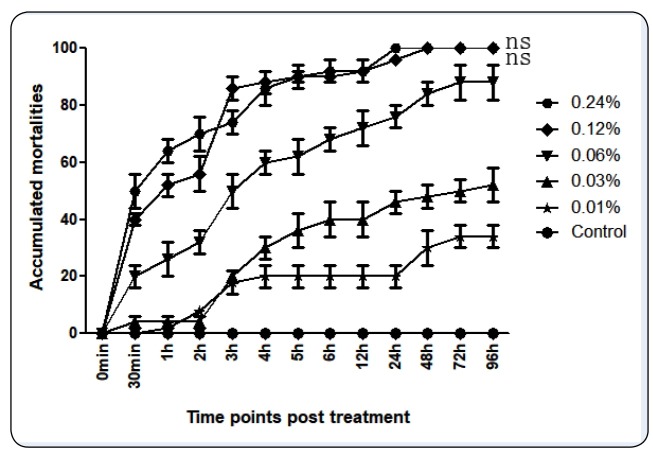
The larvicidal potentials of the *Cinnamomum camphora* (L.) essential oil against *Anopheles stephensi*. The accumulated mortality of *A. stephensi* larvae increased with the treatment time and oil concentration. There were significant differences in the accumulated mortalities among the concentration groups, based on pairwise comparisons (Mann-Whitney Rank Sum Test, P = <0.001), except for the comparison between the two groups marked “ns” (P = 0.669; P >0.05).

**FIGURE 2:**
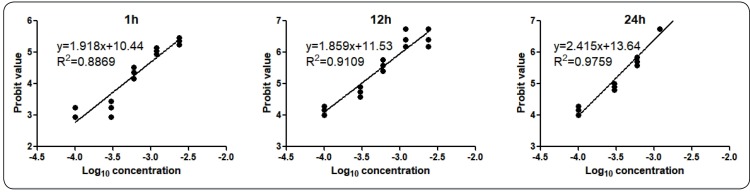
Bioassays of the killing efficiency of the essential oil against *Anopheles stephensi* larvae. Linear regression analysis of the log_10_ value of the oil concentrations and Probit value of accumulated mortalities at 1 h, 12 h, and 24 h after treatment are shown in the figures.

To explore the killing mechanisms, the chemical components of the oil were analyzed by gas chromatography-mass spectrometry (GC-MS). A GC-MS (Agilent Technologies 5975C VL MSD with triple-Axis Detector, Agilent Technologies 7890 GC System) was used to analyze the chemical composition of the essential oil from *C. camphora* (L.) leaves. DB-5MS (30.0 m × 0.25 mm × 0.25 μm) was used as the column, and a temperature at 325^o^C was used. The volatile oil sample was diluted with n-hexane at 1:30 (v/v) and the injection volume was 1 µL (flow split ratio (R) was 30:1). The carrier gas was high-purity He (99.999%) and the flow rate was 30 mL/min. The following parameters were used: ionization method, E1; electron energy, 70 eV; mass range, 30-500 m/z. As the composition of the essential oils is relatively complex, the temperature was increased during the analysis in the following manner: the initial temperature was set at 60^o^C for 1 minute, ramped to 150^o^C at a rate of 7^o^C/min, then increased to 250^o^C at a rate of 10^o^C/min, and held at 250^o^C for 15 min. The chromatographic ion chromatograms and mass spectrometry data were obtained. Based on the total ion chromatogram, the microprocessor configured by the GC-MS system was used to calculate the relative percentage of each peak area by using an area normalization method. The mass spectral data of each peak was automatically searched against the NisT standard library of the United States, and the mass fraction of each component was normalized according to the area of the peak. In addition, several published mass spectrometry datasets were analyzed to confirm the chemical compound from candidates with a similar score under one peak^4^. The GC-MS results of the essential oil are presented in Table 1. The oil of *C. camphora* (L.) contains 32 compounds, with the major components of eucalyptol (53.49%), β-terpinene (17.44%), α-terpineol (9.45%), and 1R-α-pinene (4.71%).

**TABLE 1:** Chemical composition of the essential oil of *Cinnamomum camphora* (L.) leaves.

Compound present	Retention time	% COMP
Bicyclo[3.1.0]hexane,4-methyl-1-(1-methylethyl)-,didehydro deriv.	4.848	0.70
1R-α-Pinene	5.005	4.71
L-Camphene	5.312	0.23
β-Terpinene	5.718	17.44
L-β-Pinene	5.831	3.39
β-Myrcene	5.966	1.58
α-Terpinene	6.555	0.36
p-isopropyltoluene	6.706	0.10
D-Limonene	6.814	0.58
Eucalyptol	6.922	53.49
γ-Terpinene	7.376	0.64
γ-Terpinene	7.630	0.77
Terpinolene	7.938	0.17
4-Carene, (1S,3R,6R)-(-)-	8.186	0.12
γ-Terpinene	8.267	0.37
Pseudolimonene	9.699	0.57
endo-Borneol	9.758	0.10
γ-Terpinene	9.926	1.30
α-Terpineol	10.228	9.45
Acetic acid, 1,7,7-trimethyl-bicyclo[2.2.1]hept-2-yl ester	12.022	0.16
3-Carene	13.426	0.07
β-Elemen	14.107	0.07
Caryophyllene	14.712	0.28
α-Caryophyllene	15.349	1.22
Germacrene D	15.787	0.47
α-Selinene	15.933	0.22
Calarene	16.035	0.52
Camphene	16.916	0.05
γ-Elemene	17.094	0.11
α-Guaiene	17.742	0.50
Alloaromadendrene	18.736	0.12
Alloaromadendrene	18.888	0.12

Recently, plant-sourced essential oils have attracted much attention from researchers and have been studied extensively. Raj GA et al.^7^ studied the chemical constituents of the essential oil from the seeds of *Nigella sativa* and the larvicidal activity against *Aedes aegypti*, *A. stephensi*, and *Culex quinquefasciatus*. The essential oils from salvia, *Artemisia nilagirica, Tanacetum argenteum*, and *Rosmarinus officinalis* also showed larvicidal activity against mosquitoes^8^ ^,^ ^9^ ^,^ ^10^ ^,^ ^11^. In this study, the bioassay was conducted using Probit analysis method to examine the killing effect and the mechanism of action of the essential oil from *C. camphora* (L.) leaves on the larvae of *A. stephensi*. The results showed that the essential oil from *C. camphora* (L.) leaves had a strong larvicidal activity against *A. stephensi*, with a high sensitivity and a rapid onset of action. This was therefore suggested as an alternative for the development of an effective and safe larvicide to control *Anopheles* mosquitoes. This study also provided a dose reference for the use of essential oils in the control of mosquitos. 

For further investigation of the mechanisms of action of *C. camphora* (L.) essential oil, chemical composition of the oil was determined by GC-MS analysis. As shown in the results, 32 compounds were identified. Eucalyptol was the main constituent of *C. camphora* (L.) essential oil, followed by β-terpinene, α-terpineol, and 1R-α-pinene. Eucalyptol, also known as 1,8-cineole, is a monocyclic monoterpenoid. This epoxy-monoterpene is used widely as a flavor and fragrance in consumer goods, as well as medical therapies; for example, it is used to treat airway diseases owing to its anti-inflammatory properties. Alvarez Costa et al.^12^ found that the essential oil from *Eucalyptus nitens* showed repellent and larvicidal activity against *Aedes aegypti* and *Aedes albopictus*, and that the repellent effect was not due only to the main component, 1,8-cineole. This suggested that eucalyptol (1,8-cineole) may be involved in the larvicidal activity of *C. camphora* (L.) essential oil against *A. stephensi* in conjunction with other compounds. Zhu L et al.^13^ found that the potent larvicidal compounds of *Artemisia gilvescens* essential oil against *Anopheles anthropophagus* included germacrene D, eucalyptol, and caryophyllene, which were also identified in our study. It has also been shown that the commercially available compound α-pinene had larvicidal effects against *Aedes aegypti* ^14^. Our future studies will focus on which chemical constituents of the oil are responsible for the larvicidal activity against *A. stephensi* and investigate the detailed mechanisms.

As an ideal biolarvicide, it is also necessary to further research its safety in humans and environmental impact. Natural products contain various chemical, mineral, and biological materials, which may induce mutagenicity, genotoxicity, and carcinogenicity in mammals^15^. The effects of essential oils on non-target organisms should also be investigated. In addition, for improved efficacy in practical applications, specific environmental conditions (such as water quality and temperature), mosquito density and population composition, the age of mosquitoes, and other factors should also be addressed to achieve the maximum control capacity. Finally, it is necessary to conduct an in-depth exploration of formulation development to improve the efficacy and stability and reduce costs.

We also aimed to determine if the biocidal activities of this essential oil included mosquito adulticide and mosquito repellent effects. We attempted bioassay several times with the application of essential oil in a sugar meal to test the mosquito adulticidal activity of the essential oil. No obvious killing efficacy was found in most of the tests, with only one positive result. We also tested the personal protective ability of the essential oil against mosquito bite. However, the repellent effect was not observed. In future, we will redesign our experiment to confirm the mosquito adulticidal and repellent potentials of the camphor essential oil.

In conclusion, the present study focused on the potential use of plant-sourced larvicide for mosquito control. The killing efficiency against *A. stephensi* larvae and the chemical composition of the essential oil from *C. camphora* (L.) were investigated. The findings indicated that the oil has excellent larvicidal potential for the control of *A. stephensi*, which should be helpful for the development of new, safer plant-sourced larvicides. *C. camphora* (L.) is a promising natural larvicide for controlling mosquitoes.
